# Definition and use of the variable “race” by medical students in Salvador, Brazil

**DOI:** 10.1590/S1516-31802010000400006

**Published:** 2010-07-01

**Authors:** Crésio Alves, Michele Souza Dantas da Silva, Luciana Muniz Pinto, Maria Betânia Pereira Toralles, José Tavares

**Affiliations:** I MD, PhD. Professor of Pediatrics, Department of Pediatrics, School of Medicine, Universidade Federal da Bahia (UFBA), Salvador, Bahia, Brazil.; II Medical student. Department of Pediatrics, School of Medicine, Universidade Federal da Bahia (UFBA), Salvador, Bahia, Brazil.; III Medical student. Department of Pediatrics, School of Medicine, Universidade Federal da Bahia (UFBA), Salvador, Bahia, Brazil.; IV MD, PhD. Adjunct professor, Department of Pediatrics, School of Medicine, Universidade Federal da Bahia (UFBA), Salvador, Bahia, Brazil.; V MD, PhD. Professor of Internal Medicine, Department of Medicine, School of Medicine, Universidade Federal da Bahia (UFBA), Salvador, Bahia, Brazil.

**Keywords:** Continental population groups, Ethnic groups, Biomedical research, Students, medical, Ethics, Grupos de populações continentais, Grupos étnicos, Pesquisa biomédica, Estudantes de medicina, Ética

## Abstract

**CONTEXT AND OBJECTIVE:**

The lack of a clear definition for human “race” and the importance of this topic in medical practice continue to create doubt among scholars. Here, we evaluate the use of the variable “race” by medical students in Salvador, Brazil.

**DESIGN AND SETTING:**

Cross-sectional study at a Brazilian federal public university.

**METHODS:**

221 randomly selected subjects were included. A semi-structured questionnaire was used for data collection. The results were expressed as means and standard deviations of the mean, proportions and frequencies. The χ^2^ (chi-square) test was used for the statistical calculations.

**RESULTS:**

Approximately half of the students (45.4%) used the racial group variable in their studies on clinical practice. Of these, 86.8% considered it to be relevant information in the medical records and 92.7%, important for diagnostic reasoning; 95.9% believed that it influenced the cause, expression and prevalence of diseases; 94.9% affirmed that it contributed towards estimating the risk of diseases; 80.5% thought that the therapeutic response to medications might be influenced by racial characteristics; 41.9% considered that its inclusion in research was always recommendable; and 20.3% thought it was indispensable. The main phenotypic characteristics used for racial classification were: skin color (93.2%), hair type (45.7%), nose shape (33.9%) and lip thickness (30.3%).

**CONCLUSIONS:**

Despite the importance of different racial groups in medical practice, the majority of the professionals do not use or know how to classify them. It is necessary to add to and/or expand the discussion of racial and ethnic categories in medical practice and research.

## RESUMO

**CONTEXTO E OBJETIVO:**

A falta de uma definição clara da raça humana e a importância desse tema na prática médica continua a ser fonte de dúvidas para estudiosos. No presente artigo nós avaliamos o uso da variável raça por estudantes de medicina em Salvador, Brasil.

**TIPO DE ESTUDO E LOCAL:**

corte transversal, realizado numa universidade pública federal.

**MÉTODO:**

221 estudantes, , foram incluídos. Um questionário semi-estruturado foi utilizado para a coleta dos dados. Os resultados são expressos como média e desvio-padrão da média, proporções e frequências. O teste do χ^2^ (qui-quadrado) foi utilizado para o cálculo estatístico.

**RESULTADOS:**

Aproximadamente metade dos estudantes (45,4%) usava a variável grupo racial na sua prática clínica em estudos. Desses, 86,8% a consideravam uma informação relevante no prontuário médico; 92,7% no raciocínio diagnóstico; 95,9% acreditavam que ela influenciava a causa, expressão e prevalência das doenças; 94,9% afirmaram que ela contribuía para estimar o risco de doenças; 80,5% informaram que a resposta terapêutica a medicamentos pode ser influenciada pelas características raciais; 41,9% consideravam que sua inclusão nas pesquisas era sempre recomendável; e 20,3% a avaliavam como indispensável. As principais características fenotípicas usadas para a classificação racial foram: cor da pele (93,2%), tipo de cabelo (45,7%), formato do nariz (33,9%) e espessura dos lábios (30,3%).

**CONCLUSÃO:**

Apesar de sua importância na prática médica, a maioria dos profissionais não usa e não sabe classificar os diversos grupos raciais. É necessário adicionar e/ou ampliar a discussão sobre as categorias raciais e étnicas no exercício da medicina e nas pesquisas médicas.

## INTRODUCTION

The classification of human beings according to racial group categories is one of the most controversial and most discussed themes in biology and medicine.^1,2^ According to some geneticists, from the biological point of view, human races do not exist, since they are social structures that change as time goes by. Moreover, the various definitions of race, racial group categories and racial issues indicate the complexity of the topic.^1^

Nevertheless, racial taxonomy is used as a variable of cardinal importance in studies on the prevalence and severity of diseases, therapeutic response (pharmacogenomics), identification of risk factors, indication of diagnostic tests and access to and use of health services.^2-7^ In medical schools, from the outset of physical examination classes, most students are instructed to describe patients’ names, gender, age and racial group categories in their medical histories.^1^ Indeed, racial group category is a very frequently used variable in patients’ medical files and in scientific papers, as a descriptive characteristic of the samples analyzed.

Nonetheless, the lack of a clear definition for human race and racial group categories and the real importance of this subject in medical practice and teaching and in biomedical research continue to create doubt among scholars.^1^ This is depicted by the diversity of racial classifications found in the main medical textbooks and encyclopedias, and in medical papers. In addition, this question becomes even more complex when these scientific articles do not illustrate the methodology used for racial classifications.^1^

In clinical practice, the racial classifications most frequently used are phenotypic.^8-9^ However, since appearance is determined by a small number of genes, it is not possible to make inferences regarding genetic constitution based on oligogenic traits that have been influenced by the environment.^1,8^ One of the few situations in which racial categories defined by skin color have medical importance is in dermatology (for example, basal cell carcinoma is rare among blacks, while keloids are less frequent among whites).^10^

## OBJECTIVE

In this light, the aim of the present study was to investigate the use of the variable of racial group category by the medical students at the School of Medicine of the Federal University of Bahia (Universidade Federal da Bahia, UFBA) in Salvador, Bahia, Brazil.

## METHODS

This was a cross-sectional study involving the population of students at the UFBA School of Medicine between October 2006 and April 2007. By randomization, the sample included 20% of the students from each of the 12 semester groups of the medical school. The gender and race distribution was random. A minimum of 192 students (with 95% confidence interval) was estimated as necessary to fulfill the statistical demands of the study.

Every student regularly enrolled in the second semester of 2006 in the UFBA School of Medicine was considered eligible. Refusal to participate in the study was the exclusion criterion.

The racial classification of the students was acquired through self-declaration, as well as through the evaluation of two previously trained interviewers, in accordance with the criteria established by Krieger et al.^11^ and Parra et al.^12^ The interview had a semi-structured format, in which information was sought regarding the students’ names, gender, age, marital status, origin, religion, racial group category, current semester, estimate of socioeconomic status, previous education in human sciences, participation in previous or current studies that included the variable of race or racial group category. The students’ knowledge and application of the concept of racial group in medical practice or daily life was also assessed.*

The data analysis was accomplished using the Statistical Package for the Social Sciences (SPSS, Chicago, United States), version 12.0. The descriptive analysis on the results used means and standard deviations for continuous variables and proportions for qualitative variables. The statistical inferences from the qualitative variables were made using the chi-square test. P values < 0.05 were taken to be significant with a 95% confidence interval.

This study was approved by the institution's Research Ethics Committee. Every student enrolled signed a free and informed consent statement in accordance with the recommendations of the Helsinki convention.

## RESULTS

A sample of 221 students ranging from 17 to 37 years of age, with a mean age of 23 (± 2.8) years, was enrolled in this study. There were no refusals to participate in the study. Most of the participants were male (n = 127; 57.5%) and single (n = 216; 99.7%). About half of the participants (n = 155; 70.1%) were living in a house or apartment that either they owned themselves or their families owned. More than half of them (n = 138; 62.4%) had at least one parent with a university degree.

Table 1 describes 200 (90.5%) of the students for whom a complete record of the self-declaration of racial group category and classification by the interviewers was obtained. The most prevalent self-declared racial group categories were white, mulatto and black and, if these are taken to be equivalent to the interviewers’ classification of white, mulatto (i.e. light and medium mulatto) and black (i.e. dark mulatto and black), respectively, the results from the two classifications were considered to be significantly discrepant (with chi-square of 29.05, two degrees of freedom and P < 0.00001). This was attributed to the high discrepancy among those who declared themselves to be black, since 84.4% (24.51) of the total chi-square (29.05) was due to comparison between concordant cases (dark mulatto and black) versus the discordant cases or other racial group categories (light and medium mulatto). Table 1 shows that 38% (76/200) declared themselves to be white, while the interviewers registered 38.5% (77/200) as belonging to this group. On the other hand, 13% (26/200) declared themselves to be black, while the interviewers assigning just one individual (0.5%) to this category. Nonetheless, in both classifications, the frequencies of students of African descent (mulattoes and blacks) were the same: 61.5% versus 62%. Nine students (4.5%) were classified as Oriental/Asians.

**Table 1.** Racial group classifications: self-declaration versus interviewer's evaluation

Self-declaration	Racial classification by the interviewer – n (%)						Total
	White	Light mulatto	Medium mulatto	Dark mulatto	Black	Amerindian	
White	55 (72.4)	21 (27.6)	0	0	0	0	76
Mulatto	17 (19.5)	47 (54)	21 (24.2)	2 (2.3)	0	0	87
Black	3 (11.5)	13 (50)	4 (15.4)	5 (19.2)	1 (3.9)	0	26
Oriental	1 (11.1)	5 (55.6)	2 (22.2)	1 (11.1)	0	0	9
Amerindian	1 (50)	1 (50)	0	0	0	0	2
Total	77 (38.5)	87 (43.5)	27 (13.5)	8 (4)	1 (0,5)	0	200

Concerning the predominance of racial group categories in the students’ regions of origin, 38.2% stated that they perceived a predominantly black background among their fellow countrymen; 45.9% a mulatto background; 15.0% a white background; and 4.5% an Oriental background.

When asked about the use and application of the racial group variable in their studies in the Medical School, 45.4% (n = 99/218) of the students stated that this was a subject regularly dealt with (9.6% always; 10.1% almost always; and 25.7% regularly); 33.9% (n = 74/218) stated that this subject was seldom dealt with; and 20.6% (n = 45/218) said that this racial classification was never used. From analysis on the responses according to the students’ semester, it was seen that 93.8% (n = 15/16) of the students in the first semester did not use or seldom used classifications by racial group categories, while this percentage decreased as the student advanced through the subsequent semesters. By distributing the students into the basic semesters (1^st^ to 4^th^ semesters), clinical semesters (5^th^ to 8^th^ semester) and internship semesters (9^th^ to 12^th^ semester) the frequencies of students who never or seldom used racial classifications in each of these groups were 78.3%, 47.2% and 31.7%, respectively. At the other extreme, represented by those who used almost always or always used such classifications, within these same semester groupings, the frequencies were 4.8%, 28.8% and 30%, respectively (Table 2).

**Table 2.** Use of the racial group variable in accordance with the medical students’ school semester [n (%)]

Semester	Never	Rarely	Frequently	Almost always	Always	Total
1^st^	7 (43.7)	8 (50.0)	0	0	1 (6.2)	16
2^nd^	13 (56.5)	7 (30.4)	3 (13.0)	0	0	23
3^rd^	3 (20)	10 (66.6)	1 (6.7)	1 (6.7)	0	15
4^th^	3 (10.3)	14 (48.3)	10 (34.5)	2 (6.9)	0	29
5^th^	6 (30)	6 (30)	4 (20)	2 (10)	2 (10)	20
6^th^	1 (6.7)	4 (26.7)	5 (33)	3 (20)	2 (13.3)	15
7^th^	2 (9.5)	7 (33.3)	5 (23)	3 (14.3)	4 (19)	21
8^th^	3 (18.7)	5 (31.2)	4 (25)	1 (6.2)	3 (18.7)	16
9^th^	1 (6.2)	2 (12.5)	9 (56.2)	0	4 (25)	16
10^th^	1 (6.7)	4 (26.6)	4 (26.6)	3 (20)	3 (20)	15
11^th^	2 (12.5)	4 (25)	4 (25)	5 (31.2)	1 (6.2)	16
12^th^	3 (18.7)	3 (18.7)	7 (43.7)	2 (12.5)	1 (6.2)	16
Total	45 (20.6)	74 (33.9)	56 (25.7)	22 (10.1)	21 (9.6)	218 (100)

The racial classification most used by the medical students was phenotypic, in which the following characteristics were used: skin color (93.2 %), hair type (45.7%), nose shape (33.9%), lip shape (30.3%) and, among other, less frequent traits, eye color (1.4%) and color of the forearm skin fold (0.5%).

Concerning the application of the racial group category classification in the medical students’ opinions, 86.8% considered that it was relevant to have such data in patient files; 92.7 thought that it was important for analysis and diagnostic reasoning; 95.9% believed that ethnicity had influence on the cause, expression or prevalence of diseases; 94.9% stated that knowledge about patients’ racial group categories might improve the risk estimates for some diseases; 80.5% said that the therapeutic response to drugs might be dependent on patients’ predominant racial characteristics.

Among the students (n = 29; 13.2%) who did not consider that the presence of patients’ racial classification in the files was relevant, 69.0% had never used such classifications for identifying patients or seldom did so, and 75.0% had never or seldom used such classifications in their studies within the disciplines of the medical course. Among the interviewees who considered that racial classification was a relevant item in the patient files, 48.94% believed that this variable was important in their studies (27.13% regularly; 11.17% almost always; and 10.64% always).

Analysis on the written responses from the interviewees who considered that racial classifications were important for the patient files revealed that most of these students (76.9%; n = 140/182) justified this by pointing out the statistical variations in some diseases with distinct racial group categories; 14.27% (n = 26/182) of them emphasized the relevance of this variable in clinical reasoning as a whole (regarding diagnostic suspicion, evaluation of the course of the disease and therapy); and 4.4% (n = 8/182) pointed out the auxiliary function of the racial group category variable in psychological, social and economic analyses on patients.

On the other hand, the justifications among those who did not consider that the presence of this variable in the files was of relevance were as follows: 26.3% (n = 5/19) believed that it did not change the management of the case; 21% (n = 4/19) believed that there was no consistent scientific evidence; 15.8% (n = 3/19) stated that it was impossible to classify race due to admixture; 10.5% (n = 2/19) said that most diseases were not influenced by this variable; 10.5% (n = 2/19) expressed the idea that all human beings were equal; 5.3% (n = 1/19) emphasized the mistakes in classifying race; 5.3% (n = 1/19) worried about the risk of generalizing biotypes through race (n = 1/19; 5.3%); and 5.3% (n = 1/19) said that producing racial classifications was not a role for healthcare professionals. A total of 10 people (n = 10/39) did not justified their negative responses.

Concerning the value of the racial group category variable in scientific research, 1.8% of the participants asserted that this variable does not have any role; 35.9% declared that it has importance in some situations; 41.9% considered that its inclusion in research is always recommended; and 20.3% thought that it was essential.

Asked about the possibility of a relationship between socioeconomic stratification and racial group categories, 94.0% of the interviewed believed that there was some dependence or association between these factors.

About two thirds of the interviewees (76.4%; n = 162/212) believed that there was some difference between the concepts of race and ethnicity; 13% (n = 13/212) were unable to say whether there was any difference; 17.45% (n = 37/212) considered them to be synonymous; and 4.07% (n = 9/221) did not answer this question. Among those who thought that there was a conceptual difference, most of them (48.8%; n = 79/162) explained it by saying that race was related to genetically determined biological factors, while ethnicity was associated with geographical, social and cultural factors; 19.8% (n = 32/162) did not answered the question about this difference; and 8.6% (n = 14/162) said that they did not know how to explain such a difference.

Most (61.7%) of the students between the 5^th^ and 12^th^ semesters thought that the degree of discussion about the racial group variable in the medical school was reasonable; 36.8% stated that it was not discussed thoroughly; and 1.5% believed that it was well discussed. Nevertheless, only 40% of the students affirmed that they had discussed racial classifications during activities at the Medical School. On the other hand, only 20.8% (n = 33) revealed that they had studied the criteria adopted for a racial classification method on their own.

## DISCUSSION

The human races are representations of individuals’ perceptions of other people.^13,14^ There are clear phenotypic and physiological differences among human populations. However, there is no single accepted classification or even any clear definition of race and ethnicity.^15^ While some researchers outline differences of greater objectivity for delimiting racial and ethnic classifications, others do not do so.^16^ Epidemiology relies on variables that help to differentiate populations with given health conditions, thereby providing information for science.^17,18^ The race and ethnicity of human beings are powerful tools. Nevertheless, there are prerequisites for their use in science or within society, particularly if there is a commitment towards decreasing the inequalities in healthcare.^19^

Race cannot be interpreted as or used as a synonym for ethnicity.^1^ Whereas race is defined according to physical features, geographic origin or common biological heritage, ethnicity is a concept that assimilates social, cultural, religious, linguistic and dietary variables in order to identify populations.^7^ Although 75.6% of the medical students in the UFBA School of Medicine believed that there were differences between the concepts of race and ethnicity, most of them could not explain such conceptual differences.

Most (81.9%) of the medical students were whites (39.5%) and light mulattoes (42.4%). Data from the Brazilian Institute for Geography and Statistics (Instituto Brasileiro de Geografia e Estatística, IBGE),^20^ obtained through self-declaration, show that in 2000, the population of Salvador was constituted thus: whites (23%), mulattoes (54%), blacks (20.4%), Amerindians (0.3%) and unclassified (0.68%). Although there are some limitations on making assumptions, this information shows that there was inequality relating to racial category, with regard to access to the public university system.

Several proposals aimed at establishing criteria for improving the use of the racial group variable have appeared, while others have advocated eliminating its use. For example, some authors have believed that this variable is only useful for studying patients’ racial perceptions with regard to correlation of inequalities in healthcare.^16^ Epidemiologists depend on racial classification to understand factors contributing to such diseases, such as experiences of stress due to racial prejudice or obstacles to accessing healthcare.^21,22^ In relation to this issue, most of the medical students interviewed (94.0%) stated that they believed in the possibility of a relationship between socioeconomic stratification and racial groups among the population.

In spite of these obstacles, continuous use of the race variable in the medical literature has been legitimated through its application to classification in relation to inferences about people's health, and through showing the lack of equality of social and economic opportunities.^23-25^ Therefore, at least two justifications support its use: (1) relationships between racial group, disease and social class, which are particularly important in countries like Brazil, where socioeconomic status is strongly associated with some racial groups, and where black people have higher rates of morbidity, lower life expectancy, lower access to healthcare services and sanitary systems and lower household income; and (2) relationships between diseases and phenotypes, which is corroborated by several pathological conditions, going from the higher risk of severe presentations of schistosomiasis among whites^26^ to the higher prevalence of the variant delta 32 of the CCR5 receptor among whites, which hampers the entry of the human immunodeficiency virus (HIV) into cells, thereby protecting them against infection and virus dissemination.^27^

Because of these justifications, the racial group variable continues to be used in teaching and medical research. As observed in this study, from the 4^th^ semester of the medical course onwards (when the students begin to learn the discipline of clinical examination and are taught to structure a history through describing the patient's name, gender, age and race) the number of students using and applying this variable in their studies, and in identifying patients, can be seen to increase.

In line with the medical literature, the present study revealed that in most cases, facial features were the elements used to classify race. Skin color was the main parameter for such classifications, and was chosen by 93.2% of the students. Other than this phenotypic classification, few students (5%) make use of patients’ self-declarations for racial classification, although this is the most widespread method in demographic censuses in most countries in which racial classification is recorded.^1^ In the analysis on the presence of the topic of racial classification in medical schools, 61.7% of the students from the 5^th^ to the 12^th^ semesters considered that this was reasonable. However, only 40% of them declared that this topic had been discussed in some way during the medical course.

Racial classifications in medical and epidemiological research are influenced by the country in which they are performed.^28^ In the United States, the “one drop rule” prevails, such that any person with a minimal amount of African ancestry will be considered black. Furthermore, the phenotypic classification may vary even inside a given country. For example, somebody who is classified as white in northeastern Brazil might be considered mulatto or black by an interviewer in southeastern Brazil.^1,14^ This can make these data diverge in other regions of the country, due to differences in the phenotypic perception of the racial group.

Sometimes, racial classifications make use of different concepts thereby further increasing the confusion regarding their interpretation and validation. For example, some researchers use the terms white (phenotypic classification), “Anglo” (linguistic classification) and “Caucasian” (ancestry classification) as synonyms.^1^ As is well known, this is not recommended, since skin color and other phenotypic classifications (hair type or lip and nose shape) do not equate with ancestry or ethnicity.^12^

## CONCLUSIONS

It is necessary to fine-tune the current criteria for classifying racial and ethnic categories, as well as attaining better standards, in order to use these variables in teaching, clinical practice and scientific research. Furthermore, there is an urgent need to foster the use of the racial group variable as a sociopolitical category, as a means of achieving better understanding about the composition of populations, in order to demonstrate the unevenness of access to healthcare and as a means of social justice focused on the promotion of racial equality.

## Footnotes

* The questionnaire can be sent by the authors on request.
